# Supplementary material for: Distinct patterns of SARS-CoV-2 transmission in two nearby communities in Wisconsin, USA
Source: medRxiv. 2020 Jul 10:2020.07.09.20149104. Preprint. [Version 1] doi: 10.1101/2020.07.09.20149104 (PMC7359545; doi:10.1101/2020.07.09.20149104)
Supplement: 1 [file NIHPP2020.07.09.20149104-supplement-1.pdf]

medRxiv preprint doi: https://doi.org/10.1101/2020.07.09.20149104.this version posted July 10, 2020. The copyright holder for this preprint (which was not certified by peer review) is the author/funder, who has granted medRxiv a license to display the preprint in perpetuity.
It is made available under a CC-BY 4.0 International license .

Supplemental Figures

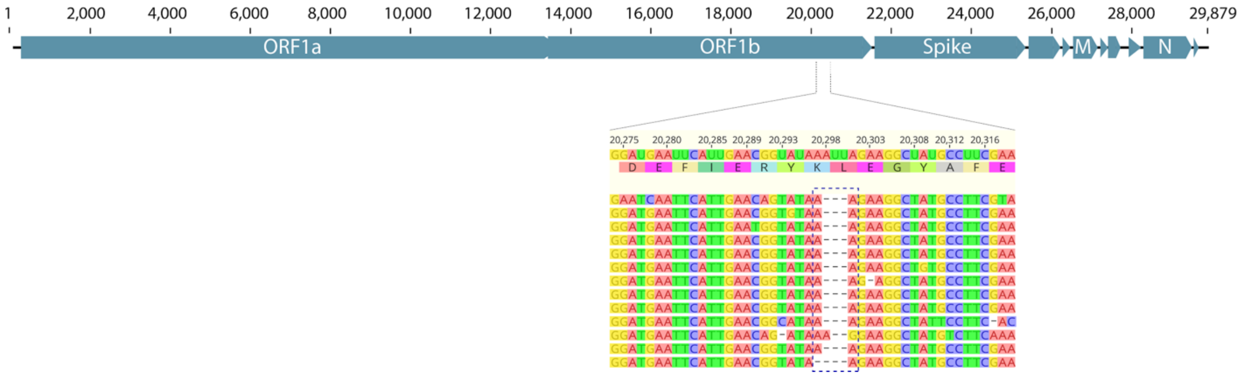

Supplemental Figure 1. Diagnostic deletion in the index Dane County sample

Consensus-level deletion identified in the Dane County index sample. Zoomed in panel shows nucleotide and amino acid identities of the in-frame deletion.

medRxiv preprint doi: https://doi.org/10.1101/2020.07.09.20149104.this version posted July 10, 2020. The copyright holder for this preprint (which was not certified by peer review) is the author/funder, who has granted medRxiv a license to display the preprint in perpetuity.
It is made available under a CC-BY 4.0 International license .

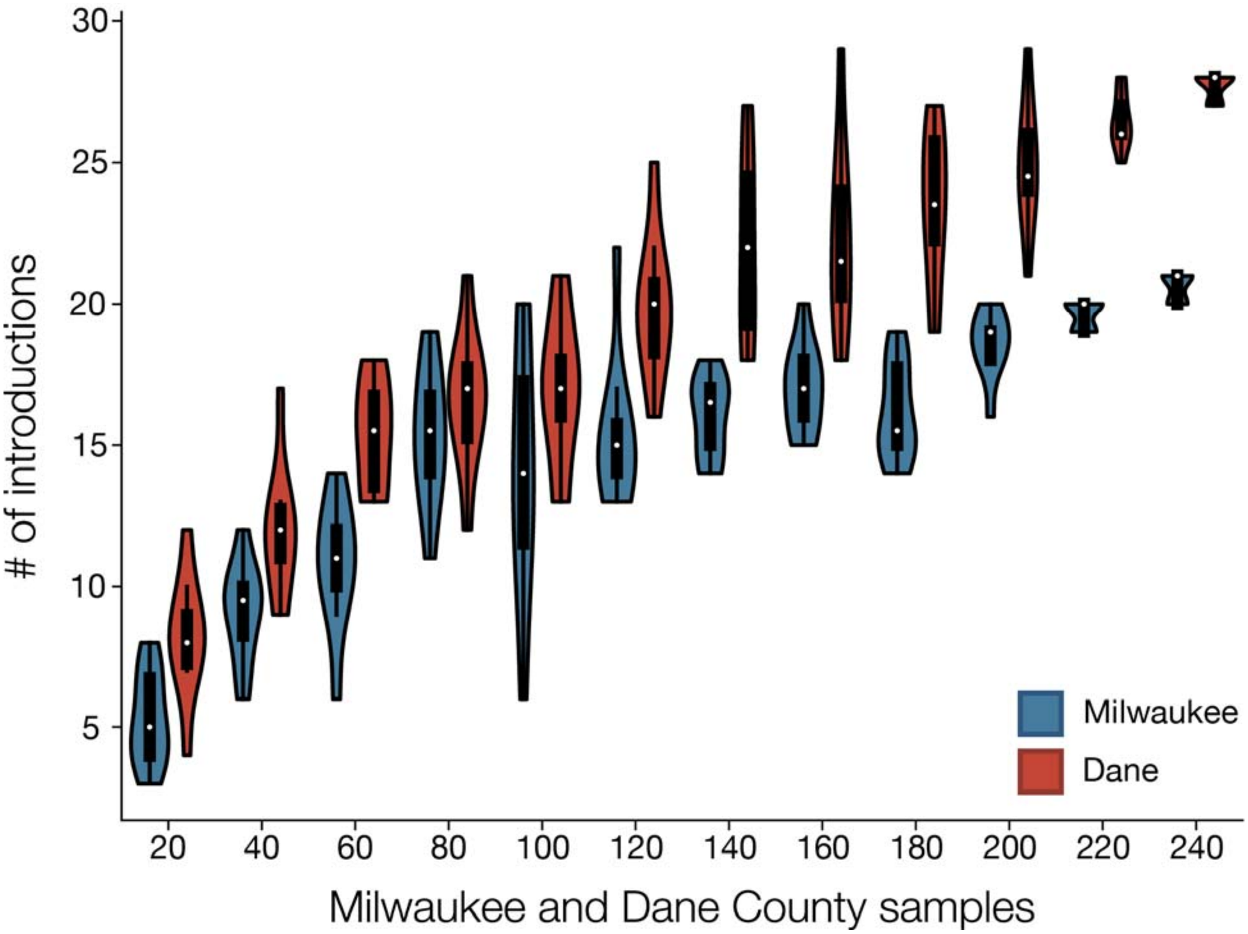

Supplemental Figure 2. Sampling sensitivity of estimates for the number of introductions into Dane and Milwaukee Counties. Estimates of the number of introductions into Dane and Milwaukee Counties using a time aligned maximum likelihood phylogeny. N sequences (x-axis) were randomly sampled from the available Dane and Milwaukee County samples and the remaining were pruned from the tree. Ten replicates of each N were conducted and the number of introductions (y-axis) was estimated for each.

medRxiv preprint doi: https://doi.org/10.1101/2020.07.09.20149104.this version posted July 10, 2020. The copyright holder for this preprint (which was not certified by peer review) is the author/funder, who has granted medRxiv a license to display the preprint in perpetuity.
It is made available under a CC-BY 4.0 International license .
